# Prevalence of *Plasmodium* spp. in the Amazonian Border Context (French Guiana–Brazil): Associated Factors and Spatial Distribution

**DOI:** 10.4269/ajtmh.19-0378

**Published:** 2019-11-25

**Authors:** Emilie Mosnier, Emmanuel Roux, Claire Cropet, Yassamine Lazrek, Olivier Moriceau, Mélanie Gaillet, Luana Mathieu, Mathieu Nacher, Magalie Demar, Guillaume Odonne, Maylis Douine, Céline Michaud, Stéphane Pelleau, Félix Djossou, Lise Musset

**Affiliations:** 1 Pôle des Centres Délocalisés de Prévention et de Soins, Centre Hospitalier Andrée Rosemon, Cayenne, France;; 2 Ecosystèmes Amazoniens et Pathologie Tropicale, EA3593, Université de Guyane, Cayenne, France;; 3 ESPACE-DEV, IRD, Université de Montpellier, Université de La Réunion, Université de Guyane, Université des Antilles, Montpellier, France;; 4 LIS, ICICT, Fiocruz, Rio de Janeiro, Brazil;; 5 Centre d’Investigation Clinique Antilles Guyane–Inserm 1424, Cayenne, France;; 6 Laboratoire de Parasitologie, Centre National de Référence du Paludisme, Pôle Zones Endémiques, WHO Collaborating Center for Surveillance of Antimalarial Drug Resistance, Institut Pasteur de la Guyane, Cayenne, France;; 7 Laboratoire de Parasitologie et Mycologie, Centre Hospitalier Andrée Rosemon, Cayenne, France;; 8 UMSR Laboratoire Ecologie, Evolution, Interactions des Systèmes Amazoniens Laboratoire Ecologie, Evolution, Interactions des Systèmes Amazoniens (LEESIA), Centre National de la Recherche Scientifique (CNRS), Cayenne, France;; 9 Unité de Maladies Infectieuses et Tropicales, Centre Hospitalier Andrée Rosemon, Cayenne, France

## Abstract

To implement future malaria elimination strategies in French Guiana, a characterization of the infectious reservoir is recommended. A cross-sectional survey was conducted between October and December 2017 in the French Guianese municipality of St Georges de l’Oyapock, located along the Brazilian border. The prevalence of *Plasmodium* spp*.* was determined using a rapid diagnostic test (RDT) and a polymerase chain reaction (PCR). Demographic, house locations, medical history, and biological data were analyzed. Factors associated with *Plasmodium* spp. carriage were analyzed using logistic regression, and the carriage localization was investigated through spatial cluster analysis. Of the 1,501 samples analyzed with PCR, positive results totaled 90 and 10 for *Plasmodium vivax* and *Plasmodium falciparum*, respectively. The general PCR prevalence was 6.6% [5.3–7.9], among which 74% were asymptomatic. Only 13/1,549 were positive by RDT. In multivariate analysis, participants older than 15 years, living in a remote neighborhood, with a prior history of malaria, anemia, and thrombocytopenia were associated with an increased odds of *Plasmodium* spp. carriage. High-risk clusters of *P. vivax* carriage were detected in the most remote neighborhoods on the village outskirts and two small foci in the village center. We also detected a hot spot for both *P. vivax* and *P. falciparum* symptomatic carriers in the northwestern part of the village. The present study confirms a wide-scale presence of asymptomatic *P. falciparum* and *P. vivax* carriers in this area. Although they were more often located in remote areas, their geographic distribution was spatially heterogeneous and complex.

## INTRODUCTION

In 2016, the incidence of malaria worldwide increased for the first time since 2000.^1^ In the Americas, this increase was largely confined to incidences in Brazil and Venezuela. French Guiana is a French overseas territory and a malaria-endemic country on the South American continent. Historically, malaria was mainly due to *Plasmodium falciparum*, but in the past 15 years, *Plasmodium vivax* infections have become predominant.^2,3^ This area is committed to a regional program for malaria control.^4^ In remote areas, diagnosis and treatment are free of charge and administered in health centers using rapid diagnostic tests (RDTs) and artemisinin combination therapies. Vector control is also conducted in all active transmission areas. In-house residual spraying is conducted in households where malaria cases are detected. In addition, permethrin insecticide–treated nets are distributed for free to pregnant women, symptomatic malaria cases, and their family members.

Although the number of malaria cases has declined over the past 10 years, the region still faces major challenges in controlling malaria transmission among socially marginalized and/or isolated populations, such as gold miners and autochthonous populations.^2,3,5,6^ Although French Guiana’s coastal and urban areas have very low malaria transmission, the country’s inland sites experience higher levels.^3,5–7^ Illegal gold miners working deep inside the rainforest are massively infected, mostly by *P. falciparum*.^5,8^ Amerindian villages along the main rivers at the borders between Suriname and Brazil represent the second largest population affected by symptomatic malaria, mostly due to *P. vivax*.^3,5,9^

Asymptomatic carriers of *Plasmodium* spp. provide a reservoir of infection in areas of transmission. Considering a parasitemia generally above 1 parasite/µL of blood, they may contribute to continuous transmission of the disease and are potentially responsible for the genesis of outbreaks.^10,11^ Therefore, to interrupt transmission, it is important to focus actions on identifying residual malaria transmission foci. Those efforts should then be intensified to eliminate any remaining foci. For *P. vivax* malaria infections, it has been clearly shown that both symptomatic and asymptomatic carriers are rapidly infectious.^12^ However, the scarcity of studies makes it difficult to identify the Amazonian region’s residual transmission areas, referred to as “malaria hotspots.” French Guiana’s population, notably gold miners, is expected to have strong malaria immunity, and many of them present asymptomatic parasitemia.^5,8,13^ Symptomatic malaria cases in this context occur throughout the year.^7^ In addition, prevalence of *Plasmodium* spp. is highly heterogeneous among the different mining sites.^5,8^ Apart from these, most of the remaining cases in French Guiana are detected at the border with Brazil (Northeastern French Guiana) and at a lower rate along the Surinamese border (Southwestern French Guiana), more specifically in Amerindian villages.^6,7^ Malaria transmission among these autochthonous populations is mostly seasonal, demonstrating an unstable pattern.^3,7^ Within these populations, it is likely that asymptomatic carriage exists.^6^

**Figure 1.**
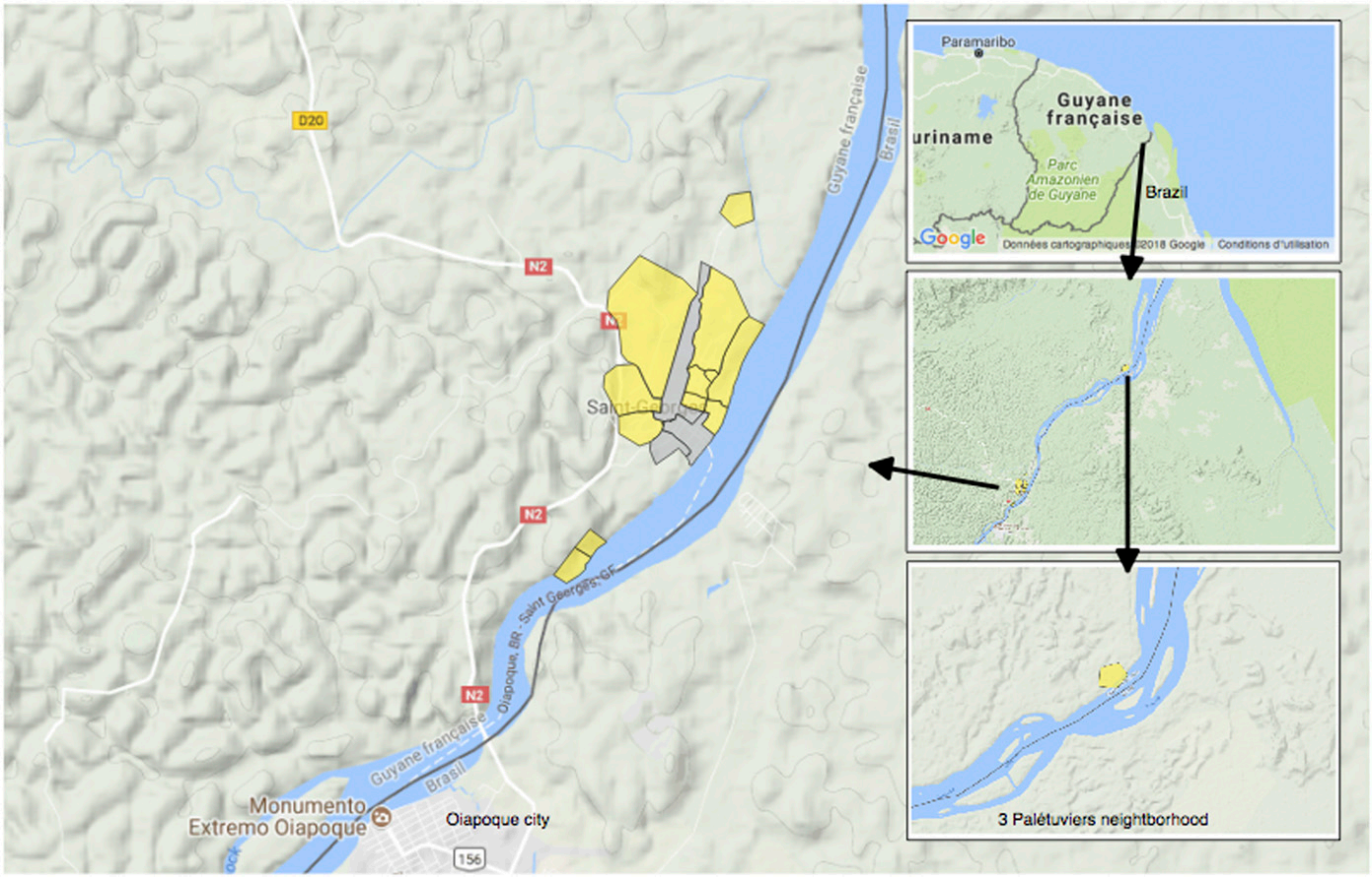
Study area. This figure appears in color at www.ajtmh.org.

This study was conducted in the village of Saint Georges de l’Oyapock (STG), a persistent malaria-endemic area along the Brazilian border (Figure 1). Two peaks in malaria cases are generally observed in this region and are consistent with the equatorial climate. These peaks, occurring in June and November, are inter-seasonal and observed between the rainy and the dry seasons.^7^ In this municipality, *Anopheles Darlingi* was previously described as the predominant species.^14^ The vector density and number of breeding sites are high beginning a few weeks before the onset of symptomatic cases.^14,15^ However, the density of *An. darlingi* is heterogeneous, depending notably on the distance to the dense forest.^16^

To date, no study has investigated the distribution of *Plasmodium* spp. carriage in this Amazonian area, and the risk factors associated with malaria parasite carriage remain unclear. The aim of this study was to provide a better understanding of the distribution and contribution of *Plasmodium* spp. carriage to the overall parasite reservoir.

## METHODS

### Study area.

This transversal study was carried out between October and December 2017 in the municipality of STG (Figure 1), and was the first part of a before–after study called “Palustop.” It was conducted to evaluate the efficiency in treating both symptomatic and asymptomatic *Plasmodium* spp. carriers to decrease malaria prevalence in the study area. This period of the year was chosen to cover the epidemic peak observed annually in this municipality.

To characterize the risk factors for malaria and the prevalence of *Plasmodium* spp. infection in this transmission area, 13 STG neighborhoods were selected based on their history of an elevated incidence of symptomatic cases over the last 2 years. These neighborhoods also included three remote villages located in the south of the municipality, 10 minutes by canoe for Blondin, or in the north, 1 hour by canoe for Trois-Palétuviers (Figure 1).

### Sample size and population.

There are approximately 4,033 inhabitants (STG health center data 2017) in STG, mainly in the municipality center. Three neighborhoods (Blondin 1, Blondin 2, and Trois-Palétuviers) are particularly isolated and only accessible by canoe. The entire population size of the 11 studied neighborhoods was ∼2,727 inhabitants, all ages and nationalities included.

Designed for the “Palustop” before–after study, the sample size was calculated at 1,200 (expected polymerase chain reaction [PCR] prevalence of 4% based on previous reported data, error margin, and an alpha risk of 0.05).^6^ Because of the good participation and mobility of the population in this area, we targeted a sample size of 1,500 participants for the first step of the study, expecting to finish a year later with a minimum of 1,200.^6^

### Biological analysis.

Malaria RDTs were performed to immediately treat participants who tested positive. We applied the most commonly used RDT in French Guiana, the SD BIOLINE^®^ Malaria Ag Pf/Pan test (pfHRP2/pLDH-based Standard Diagnostics, SD Bioline Standard Diagnostics, Gyeonggi, Republic of Korea). Malaria PCR detection was performed using a real-time method derived from Shokoples et al*.*^17^ Detection and identification of four *Plasmodium* species (*P. falciparum*, *P. vivax*, *Plasmodium ovale*, and *Plasmodium malariae*) were performed using a Taqman^®^ (Vilnius, Lithuania) probe strategy, with a sensitivity of 1 parasite/µL for all the species except *P. vivax* at 0.5p/µL. Positive and negative controls were systematically added to the experiments. This method is certified according to the International Organization for Standardization 15189 requirements for medical examinations. Complete blood count, reticulocyte, and the glucose-6-phosphate dehydrogenase (G6PD) activity tests were also performed.

### Interviews and medical care.

Participants were interviewed using a questionnaire designed to gather information on knowledge, attitudes, and practices regarding malaria. Data on demographic factors such as gender, age, and occupation were collected. Participants were also questioned about their travel habits such as frequency and location of hunting, fishing, and farming in the border area. For children, parental consent was obtained and parents were interviewed on behalf of the child. The questionnaire was administered by trained cultural mediators in the language of the participants. Their houses were georeferenced. Participants’ body temperatures were measured by an ear thermometer, and fever was defined as any temperature ≥ 38°C. All participants received a medical examination. If RDT or PCR results were positive, participants were treated according to national treatment guidelines.^18^

### Data analysis.

Symptomatic cases were defined as RDT- or PCR-positive results associated with a history of fever in the last 48 hours or during the inclusion process. *Plasmodium* spp. carriage was defined as a *Plasmodium* PCR-positive result, regardless of body temperature.

#### Spatial statistics.

Bernoulli methods proposed by Kulldorff et al.^19^ were applied via SaTScan software (Monte-Carlo method using 999 replications, version 9.5). Clusters of high or low rates with maximum likelihood ratios were selected, and any overlapping clusters were excluded. A multi-scale analysis was performed by considering the study area as a whole (study area scale). Then, because of their relative distances, the neighborhoods of Trois-Palétuviers, Blondin (1 and 2), and the STG downtown area were considered separately (locality scale). Different analyses were also performed according to the *Plasmodium* species (all species, *P. vivax*, or *P. falciparum*) and the presence or absence of symptoms (symptomatic and asymptomatic cases, symptomatic cases only, and asymptomatic cases only). Map analyses were conducted in QGIS 2.12 software.

#### Risk factor analysis.

Associated factors of *Plasmodium* spp. carriage were investigated using logistic regression models. Bivariate analyses were used to select potential covariates (*P* < 0.1) for the multivariate model. A backward selection procedure was then applied to keep only significant associated factors (*P* < 0.05) in the final multivariate model. Odds ratios and associated 95% CIs were estimated. Statistical analyses were conducted with SAS version 9.4 software (SAS Institute Inc., Cary, NC).

### Ethics approval and consent to participate.

The study was approved by the *Comité de Protection des Personnes du Sud-Ouest et Outre-Mer 4* N° AM-36/1/CPP15-024 (French ethics committee). The database was anonymized and declared to the French Regulatory Commission (Commission Nationale Informatique et Libertés, CNIL, n°917186). Samples collected by the National Reference Center were registered by the French Ministry for Research (declaration number DC-2010-1223; collection N°2). Duly signed, informed consent was obtained from all participants involved in the study, following explanations about the study in their local languages. Treatments and medical follow-up of malaria infection were offered.

## RESULTS

A total of 1,566 inhabitants were included in the study (57.4% of the study area inhabitants). Of these, 1,501 (95.8%) individuals had a blood sample taken. Venipuncture failure or fear of blood sampling was the primary reason why certain participants did not contribute to the blood samples. The median age was 22.8 years (min–max: 0.2–92.9) and the sex ratio was 0.88 (Table 1). A majority of participants declared having French nationality (56.7%), 42.7% Brazilian, and 0.6% another nationality (Table 1). The study population was multiethnic, with more than 12 different native languages reported. A third (32.8%) of the participants older than 18 years never went to school or reported having only a primary level of education. A large portion (45.2%) of the participants reported living in the same house with a large group of individuals (more than six people). A minority (18.5%) of participants did not have social coverage or reported having precarious social protection.

**Table 1:** Main characteristics of study participants, STG, 2017

Patients characteristics (*n* = 1,566)	Number or median	% or min–max
Age (years)	22.8	(0.2–92.9)
Age categories (years)		
0–14	710	45.3
15–24	232	14.8
≥ 25	624	39.8
Male	735	47
Level of education if age > 18 years (*n* = 786)		
No formal education	122	15.5
Primary	136	17.3
College	318	40.4
High school	169	21.5
University	41	5.2
Occupation		
Farmer	111	7.1
Hunter	38	2.4
Work at home	333	21.3
Student	572	36.5
Gold miner	1	0.06
Fisherman	28	1.8
Canoe driver	4	0.3
Pensioner	37	2.4
Employee in the center of STG	88	2.6
Employee in the center of Oiapoque city	5	0.3
Others	349	22.3
Number of people in a household (*n* = 1,562)		
1–3	244	15.6
4–6	612	39.2
6–10	529	33.9
Greater than 10	177	11.3
Nationality		
French	888	56.7
Brazilian	668	42.7
Surinamese	3	0.3
Haitian	2	0.1
Guyanese	1	0.06
Other	4	0.3
Native language		
Brazilian	513	32.8
French	98	6.3
Creoles from French Guiana	371	23.7
Creoles from Haiti	4	0.3
Palikur	372	23.7
Karipuna	70	4.5
Kalina	2	0.1
Wayampi	15	0.9
Teko	39	2.5
Others	82	5.2
Social coverage		
French State Medical Assistance*	144	7.3
French Universal Health Coverage	1,089	69.5
Brazilian social coverage	57	3.6
French social security	79	5
No social coverage	175	11.2
Unknown	52	3.3
Sleeping under bednets		
No	408	26
Yes	1,558	74
Medical history of confirmed malaria†		
No	846	54
Yes	720	46
If age older than 18 years		
No	241	31.8
Yes	517	68.2
Number of episodes of malaria (*N* = 720)	3.0	(2.8–3.6)
Year of last episode		
2017	132	18.3
2014–2016	123	17
2000–2014	358	49.7
≤ 2000	107	14.9
Malaria species of the last episode		
*Plasmodium falciparum*	94	13
*Plasmodium vivax*	394	54.7
Unknown	226	31.4
Other	6	0.8

STG = Saint Georges de l’Oyapock.

* State Medical Assistance: Social coverage for an immigrant without a residency permit or a document proving that the immigrant has begun the application process for legal residency.

† Malaria confirmed by a test in a health center.

### Common biological characteristics.

**Figure 2.**
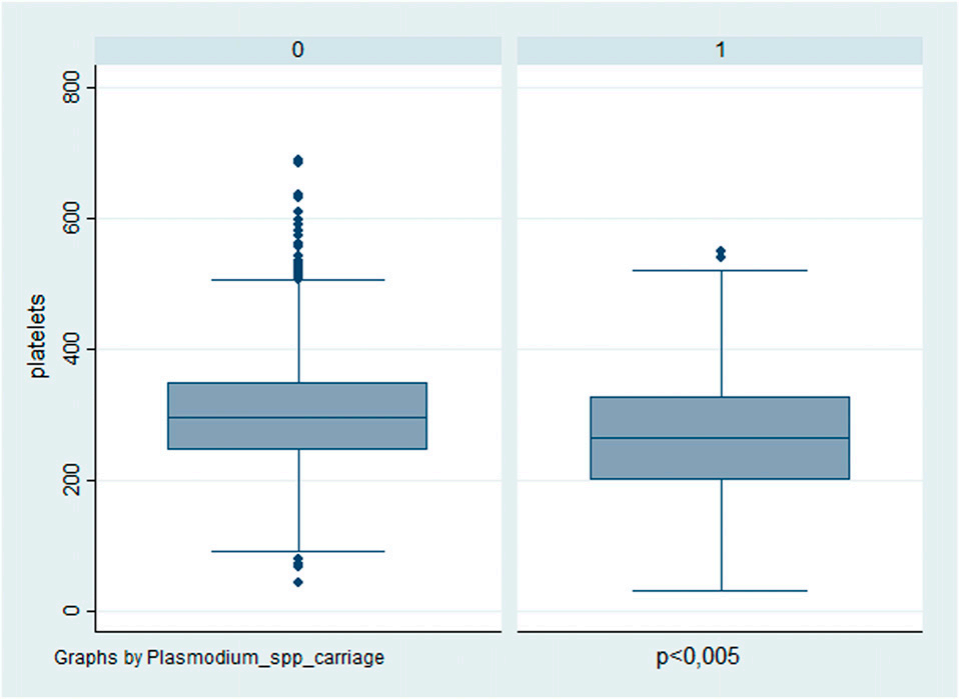
Level of platelets in participants with or without *Plasmodium* spp. carriage. This figure appears in color at www.ajtmh.org.

Severe anemia (< 8g/dL) was found in only two participants (0.14%). Anemia (< 10g/dL) and thrombocytopenia (< 150 10^9^/L) were associated with *Plasmodium* spp. carriage (Table 2 and Figure 2). Hemolysis was uncommon; only 34 participants (2.3%) had more than 2.5% of reticulocytes. Participants (*n* = 1,470) were screened for G6PD deficiency (*N* > 10 U/g Hg). The median value of G6PD was 11.9 [11.7–12.2] in males and 12.2 [12.0–12.5] in females. According to the WHO classification for G6PD activity ranges, the majority of them (91.2%, *n* = 1,345/1,474) had a normal G6PD activity (> 80%), 67 females (4.5%, *n* = 67/1,474) had an intermediate activity (30–80%), and only three females (0.2%, *n* = 3/1,474) and one male (0.06%, *n* = 1/1,474) had an activity less than 30% and 10%, respectively.^20^

**Table 2:** Factors associated with *Plasmodium* spp*.* carriage based on PCR results in univariate analysis, STG, 2017

	*Plasmodium* spp. PCR-negative (*n* = =1,401)	*Plasmodium* spp. PCR-positive (*n* = =100)	*P*-value
Age (years)†	23.5 (22.7–24.73)	26.63 (23.6–30.19)	0.04
≤ 14	639 (95.7%)	29 (4.3%)	*<* 0.005
15–24	197 (89.1%)	24 (10.9%)	
25–49	410 (92.1%)	35 (7.9%)	
≥ 50	155 (92.8%)	12 (7.2%)	
Gender			0.624
Female	750 (93.6%)	51 (6.4%)	
Male	651 (93.0%)	49 (7.0%)	
Nationality			0.943
French	784 (93.2%)	60 (6.8%)	
Brazilian	607 (93.8%)	40 (6.2%)	
Guyanese	1 (100.0%)	0 (0.0%)	
Haitian	2 (100.0%)	0 (0.0%)	
Surinamese	3 (100.0%)	0 (0.0%)	
Others	4 (100.0%)	0 (0.0%)	
Mother tongue†			*<* 0.005
French	90 (97.8%)	2 (2.2%)	
Creole from French Guiana	315 (88.0%)	43 (12.0%)	
Brazilian	465 (94.3%)	28 (5.7%)	
Palikur Indians	344 (95.3%)	17 (4.7%)	
Karipuna Indians	63 (94.0%)	4 (6.0%)	
Teko or Wayãpi Indians	51 (98.0%)	1 (2.0%)	
Saramaka (Maroons)	0 (0.0%)	1 (100.0%)	
Others	73 (94.8%)	4 (5.2%)	
Amerindian or creole ethnicity*			0.030
No	208 (96.7)	7 (3.3%)	
Yes	1,193 (92.8%)	93 (7.2%)	
School level			0.179
Any level	249 (94.3%)	15 (5.7%)	
Nursery school	106 (95.5%)	5 (4.5%)	
Elementary school	368 (93.9%)	24 (6.2%)	
High school	178 (89.0%)	22 (11.0%)	
University	38 (92.7%)	3 (7.3%)	
Occupation†			0.023
Farmers	99 (90.0%)	11 (10.0%)	
Hunters	31 (83.8%)	6 (16.2%)	
Canoe driver or fisherman	27 (84.4%)	5 (15.6%)	
Occupation in downtown area	85 (93.4%)	6 (6.6%)	
Student	526 (95.1%)	27 (4.9%)	
Work at home (housewife notably)	309 (93.9%)	20 (6.1%)	
Others	324 (92.8%)	25 (7.2%)	
Residence area†			*<* 0.005
Blondin 1 and 2 neighborhoods	42 (73.4%)	13 (23.6%)	
Trois-Palétuviers neighborhood	133 (73.9%)	47 (26.1%)	
Other neighborhood (village center)	1,266 (96.9%)	40 (3.1%)	
Time spent in STG (years)			0.799
< 2	164 (93.2%)	12 (6.8%)	
2–4	187 (94.4%)	11 (5.6%)	
≥ 4	1,050 (98.0%)	77 (2.0%)	
Mean of people in a household (*n* = 1,497)	6.4 (6.2–6.5)	7.6 (6.8–8.3)	*<* 0.005
Bednets use†			*<* 0.005
No	374 (96.4%)	14 (3.6%)	
Yes	1,027 (92.3%)	86 (7.7%)	
Bednets with holes (*n* = 1,073)			*<* 0.005
Yes	666 (90.6%)	69 (9.4%)	
No	322 (95.3%)	16 (4.7%)	
Medical history of malaria†			< 0.005
Yes	769 (97.5%)	20 (2.5%)	
No	632 (88.8%)	80 (11.2%)	
Complete antimalarial treatment during the last event (*n* = 712)			0.48
Yes	42 (87.7%)	7 (14.3%)	
No	590 (89.0%)	73 (11.0%)	
Use traditional plants†			< 0.005
Never	913 (94.7%)	51 (5.3%)	
Sometimes	387 (90.6%)	40 (9.4%)	
Often	110 (92.4%)	9 (7.6%)	
Hunting practice†			< 0.005
Yes	221 (89.1%)	27 (10.9%)	
No	1,180 (94.2%)	73 (5.8%)	
Fishing practice†			< 0.005
Yes	385 (88.9%)	48 (11.1%)	
No	1,016 (87.0%)	52 (3.0%)	
Farming activity in the rain forest†			< 0.005
Yes	639 (90.1%)	70 (9.9%)	
No	762 (96.2%)	30 (3.8%)	
Gold mine activity or spent time in a gold mine†			0.021
No	1,356 (93.6%)	92 (6.4%)	
Yes	45 (84.9%)	8 (15.1%)	
History of fever in the last 48 hours†			< 0.005
Yes	115 (81.6%)	26 (18.4%)	
No	1,286 (88.1%)	74 (11.9%)	
Temperature†			0.007
< 38	1,379 (93.6%)	94 (6.4%)	
≥ 38	21 (77.8%)	6 (22.2%)	
Physical examination†			0.003
Normal	1,073 (93.9%)	62 (6.1%)	
Not normal	327 (89.6%)	38 (10.4%)	
Missing data	1 (100.0%)	0 (0.0%)	
Rapid diagnostic test†			< 0.005
Negative	1,390 (93.9%)	91 (6.1%)	
Positive	4 (30.8%)	9 (69.2%)	
Missing data or cannot be interpreted	7 (100.0%)	0 (0.0%)	
Hemoglobin count (g/dL)†			< 0.005
< 10	16 (72.7%)	6 (27.3%)	
≥ 10	1,358 (93.5%)	94 (6.5%)	
Missing data	27 (100.0%)	0 (0.0%)	
Platelets count†			< 0.005
< 150 10^9^/L	24 (63.2%)	14 (36.8%)	
≥ 150 10^9^/L	1,350 (94.0%)	86 (6.0%)	
Missing data	27 (100.0%)	0 (0.0%)	
Eosinophil count (G/L)			0.439
≤ 0.5	668 (93.4%)	47 (6.6%)	
> 0.5	675 (94.4%)	40 (5.6%)	
Missing data	58 (56.3%)	13 (43.7%)	
Glucose-6-phosphate dehydrogenase activity			0.793
< 80%	120 (93.8%)	8 (6.2%)	
≥ 80%	1,249 (93.1%)	92 (6.9%)	
Missing data	32 (100.0%)	0 (0.0%)	

PCR = polymerase chain reaction; STG = Saint Georges de l’Oyapock.

* Wayana, Palikur, Kalina, Wayãpi, Teko, and Karipuna Amerindian communities were grouped on one side, Haitian and French Guianese Creole on the other side.

† Variable used in multivariable analysis.

### *Plasmodium* spp. carriers.

**Figure 3.**
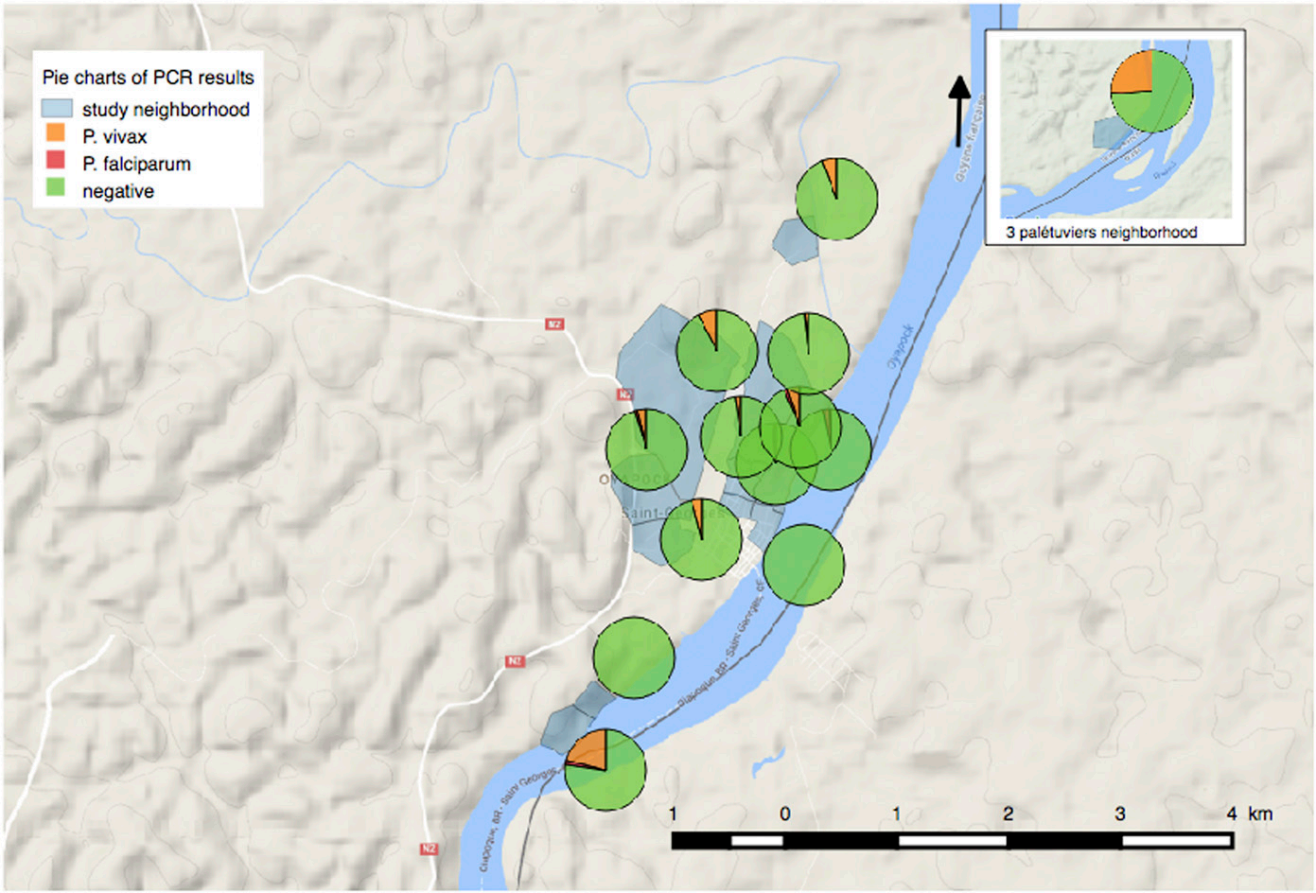

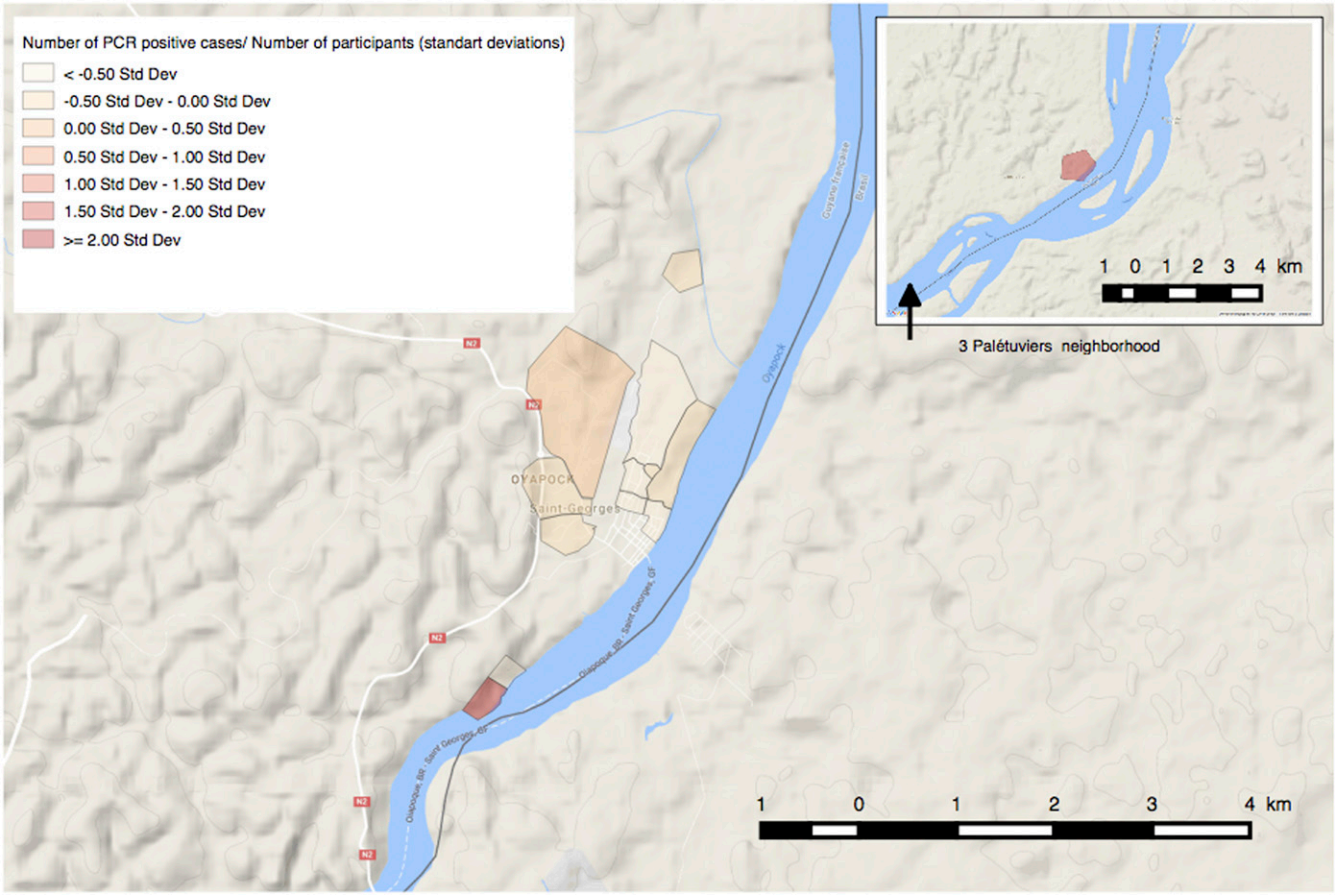
*Plasmodium* spp. polymerase chain reaction (PCR) results by neighborhood and number of PCR-positive results per study participant. This figure appears in color at www.ajtmh.org.

**Figure 4.**
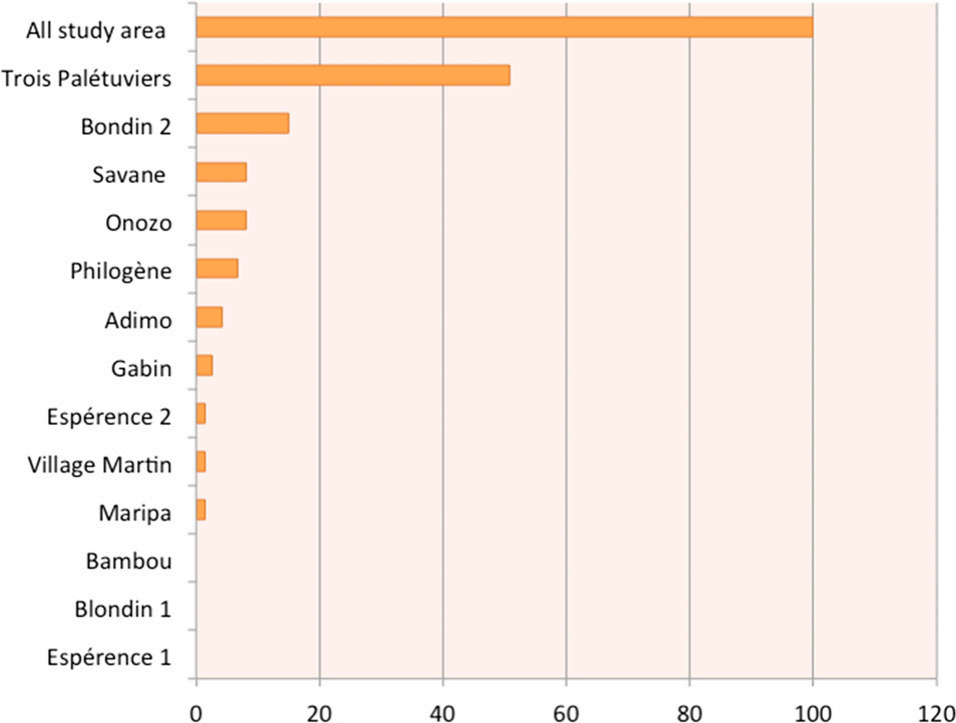
Percentage of asymptomatic *Plasmodium* spp. carriage by neighborhood. This figure appears in color at www.ajtmh.org.

The prevalence of *Plasmodium* spp. carriers was 6.6% [5.3–7.9], with the 100 positive cases being predominantly *P. vivax* (*n* = 90). In comparison, malaria prevalence using RDTs was 0.84% ([0.38–1.3], *n* = 13/1,540). The median age of the PCR-positive population was 23.5 years (min–max: 0.8–74.3), significantly higher than that of the PCR-negative population (*P* = 0.04, Table 2). Asymptomatic carriers represented 73.0% (*n* = 73/100) and 38.5% (*n* = 5/13) of the PCR- and RDT-positive population, respectively. Asymptomatic carriage also varied according to the *Plasmodium* species: 100% (*n* = 10/10) for *P. falciparum* and 70.0% (*n* = 63/90) for *P. vivax* (*P* < 0.005). A strong heterogeneity of *Plasmodium* spp. carriage between neighborhoods ranged from 0.0% to 29.5% (Table 3, Figures 3 and 4).

**Table 3:** *Plasmodium* spp*.* prevalence per neighborhood, STG, 2017

STG neighborhoods	Results of polymerase chain reaction				Studied participants (*n*)	Exhaustivity*
	*Plasmodium* spp*.* prevalence % (IC 95%)	Negative	*Plasmodium vivax*	*Plasmodium falciparum*		
Total	6.4 (5.3–7.9)	1,401	90	10	1,566	57.4%
Blondin 2	29.5 (15.9–43.2)	31	12	1	44	91.7%
Trois-Palétuviers	26.1 (19.6–32.5)	133	46	1	183	98.9%
Adimo	7.5 (2.4–12.6)	98	8	0	111	41.7%
Philogène	6.5 (0.9–12.0)	72	4	1	77	90.6%
Village Martin	5.7 (2.0–13.5)	33	2	0	35	42.3%
Gabin	4.7 (0.6–8.7)	102	4	1	113	78.2%
Maripa	3.9 (1.4–9.3)	49	2	0	54	38.6%
Onozo	3.0 (0.8–5.3)	221	2	5	252	53.5%
Esperance 2	2.4 (0.3–5.0)	124	3	0	137	44.1%
Savane	1.7 (0.4–2.9)	409	6	1	425	65.4%
Esperance 1	1.3 (1.3–3.9)	75	1	0	79	67.5%
Bambou	0.0	43	0	0	45	24.2%
Blondin 1	0.0	11	0	0	11	68.7%

IC = interval confident; *n* = number; STG = Saint Georges de l’Oyapock.

* Total numbers of inhabitants per neighborhood (*n* = 2,727) were derived from the STG health center data to calculate the exhaustivity level, 2017.

In bivariate analysis, asymptomatic carriage was more common in participants older than 15 years as well as in the Amerindian and Creole communities (Table 2). Autochthonous practices such as hunting, fishing, farming, and using traditional plants were also associated with asymptomatic malaria carriage (Table 2). In bivariate analysis, sleeping under bednets and the presence of holes in bednets among those who reported using them were related to a higher *Plasmodium spp*. prevalence. Previous history of malaria was associated with *Plasmodium* spp. carriage.

### Spatial distribution of *Plasmodium* spp*.* carriers.

Spatial data from thirteen individuals, all associated with negative PCR results, were removed from the analysis because of either missing (*n* = 5) or erroneous (*n* = 8) global positioning system localizations.

**Figure 5.**
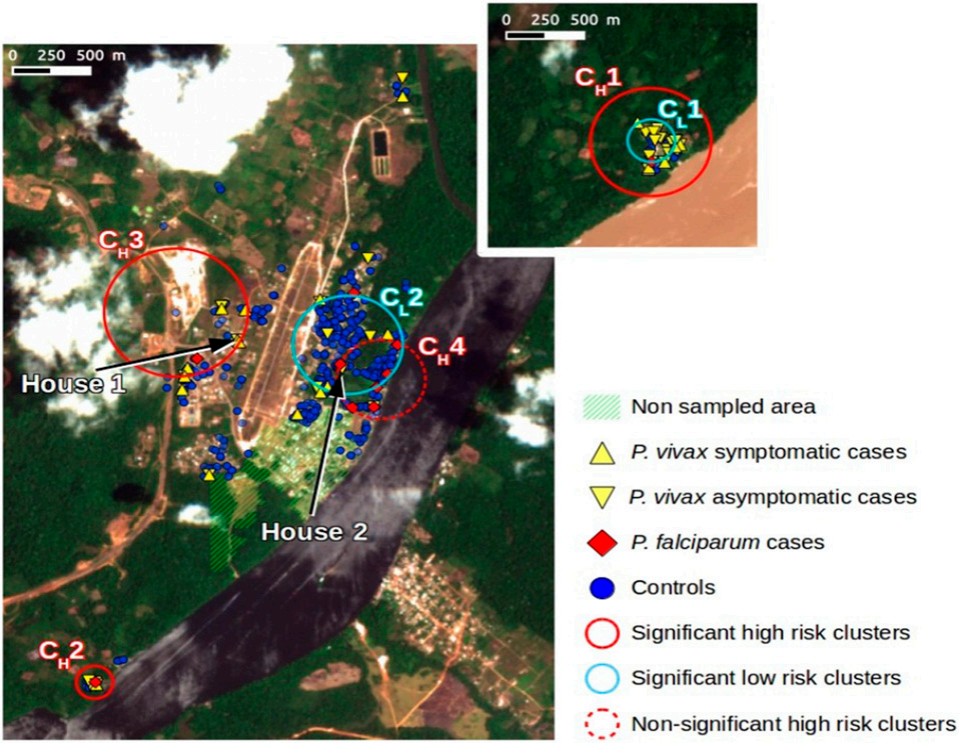
Results summary of spatial clustering using multiple outcomes. This figure appears in color at www.ajtmh.org.

At the whole study area scale, the Trois-Palétuviers neighborhood alone formed a high-risk cluster for asymptomatic *P. vivax* cases (*P* < 0.005, see cluster CH1 in Figure 5). The relative risks were 6.97, 8.21, and 9.29 for the positive *Plasmodium* spp. cases, *P. vivax* cases only (asymptomatic and symptomatic), and *Plasmodium* spp. asymptomatic cases, respectively.

The study of the Trois-Palétuviers neighborhood alone (locality scale) indicated that the “center” of the neighborhood formed a low-risk cluster (see Figure 5, cluster CL1) (RR=0, *P*-value = 0.038) for symptomatic cases (considering *Plasmodium* spp.).

At the whole study area scale, the southern part of the Blondin neighborhood (Blondin 2) formed a high-risk cluster for *P. vivax* asymptomatic cases also (see cluster CH2 in Figure 5). The relative risks were 6.63 (*P* < 0.005), 6.67 (*P* < 0.005), and 10.71 (*P*-value = 0.0002) for the positive *Plasmodium* spp. cases, *P. vivax* cases only (asymptomatic and symptomatic), and *Plasmodium* spp. asymptomatic cases, respectively. No other significant results were derived from the local scale analysis for this neighborhood.

Two high-risk clusters in the Saint Georges downtown area corresponded, in fact, to specific homes (denoted as House 1 and House 2 in Figure 5). One house was located in the Adimo neighborhood, which counted four *P. vivax* carriers (two asymptomatics and two symptomatics) of the five household members. The second significant high-risk cluster, located in the Philogène quarter, included two neighboring houses, which had four asymptomatic *P. vivax* carriers among the eight household members. Except for these two very small hot spots, the surrounding neighborhoods presented significantly low relative risks. One of the most significant (*P* < 0.005) low-risk clusters (RR = 0.14), denoted as CL2, is represented in Figure 5.

By considering symptomatic cases for *Plasmodium* spp. at the local scale, a significant high-risk cluster was identified in the northwestern part of the village’s urban area (the western part of the Adimo quarter and the northern part of the Gabin quarter; see cluster CH3 in Figure 5). The associated relative risk was 10.82 (*P* = 0.046). All the symptomatic cases in this area were *P. vivax* cases. What is more, among the total number of cases in this cluster (12), only one was associated with *P. falciparum*. Thus, these results reveal that this cluster predominantly comprised symptomatic *P. vivax* cases.

*Plasmodium falciparum* cases were pooled in the eastern part of the Saint Georges locality (CH4 in Figure 5). However, this cluster was not statistically significant (*P* = 0.417).

### Analysis of factors associated with *Plasmodium* spp. carriage.

Independent associated factors of *Plasmodium* spp. carriage are shown in Table 4. The main factors were identified as “living in an outlying neighborhood” (Blondin or Trois-Palétuviers), “being older than 14 years,” “having reported a previous history of malaria,” and “having reported a history of fever in the last 48 hours.” Anemia and thrombocytopenia were also found to be associated with *Plasmodium* spp*.* carriage in the final multivariate model.

**Table 4:** Risk factors for *Plasmodium* spp*.* carriage in multivariable analysis, Saint Georges de l’Oyapock, 2017

Variable		Odds ratio	95% Wald confidence limits
Neighborhood	Trois-Palétuviers vs. downtown	13.40	7.86–22.88
	Blondin 2 vs. downtown	9.85	4.51–21.88
Age (years)	[15–24] vs. ≤ 14	2.49	1.64–4.89
	≥ 25 vs. ≤ 14	1.86	1.05–3.30
Medical history of malaria	Yes vs. no	2.66	1.05–4.72
History of fever in the last 48 hours	Yes vs. no	3.66	1.99–6.74
Anemia (g/dL)	< 10 vs. ≥ 10	10.38	3.11–34.57
Thrombocytopenia	< 150 10^9^/L vs. ≥ 150 10^9^/L	6.87	2.87–16.45

## DISCUSSION

Active case detection of malaria in this border area revealed a heterogeneous *Plasmodium* spp. prevalence, ranging from 0.0% to 29.5% depending mainly on the neighborhood. The general prevalence was 6.6% IC_95%_ (5.3–7.9). The majority carried *P. vivax* (90.0%) and was asymptomatic (74.0%).

### Specificity of *Plasmodium* spp. carriage in this population.

Numerous behavioral factors can increase the risk of infection. The present study showed that people aged from 14 to 25 years formed the most significant at-risk group. People older than the age of 14 may have more at-risk behaviors such as night hunting or fishing. Other contributing factors include activities in high transmission sites such as gold mines or even staying outside with friends during sunset, a known *Anopheles*-vector biting time (4). The present study surprisingly revealed that the use of bednets is associated with *Plasmodium* spp. carriage in bivariate analysis. Also, when bednets were used, the presence of holes was associated with *Plasmodium* spp. carriage. In the same way, participants who reported the use of plants for medicinal purposes had a higher *Plasmodium* spp. carriage prevalence. This may be due to a confounding bias: Amerindian and Creole communities were the hardest hit by malaria and had the greatest numbers of users of traditional medicine and bednets.

### Heterogeneous *Plasmodium* spp. prevalence in the same village.

As the current global context moves toward malaria elimination, shifts toward heterogeneous transmission of the disease are generally observed.^21,22^ The situation in this part of French Guiana reflects a similar trend. Blondin 2 and Trois-Palétuviers, the most remote neighborhoods, appeared to be the largest hot spots. Indeed, these results indicated that living near the forest and vector-breeding sites is associated with elevated *Plasmodium* spp. carriage, as previously identified along the Oyapock River.^14,23^ Concordantly, in the Trois-Palétuviers neighborhood, symptomatic cases were reported more significantly in the outlying areas, near high vector density sites. The role of vector density and human behaviors in sustaining symptomatic cases in this micro-area deserves to be explored. It is worth noting that complex malaria transmission foci were also found in the STG village center within Amerindian families. In this context, travel or certain practices (frequent fishing and hunting expeditions in this community) would explain those cases. Similarly, most *P. falciparum* carriage appeared in Onozo, a fishing neighborhood that may have hosted gold miners. Determining additional risk factors may help guide effective control programs among these border populations. Such risk factors include travel to specific regions or villages linked to imported malaria cases and potentially resulting in the start of transmission chains.^24^ In addition, the present study describes a fine-scale spatial distribution of *Plasmodium* spp. carriage, but further studies are needed to determine whether or not these hot spots are stable and potentially responsible for the seasonal transmission observed each year.

### A majority of asymptomatic carriage with frequent biological abnormalities.

*Plasmodium* spp. carriage, notably asymptomatic malaria, has significant health consequences. For example, anemia and thrombopenia are observed in both symptomatic and asymptomatic infections. Asymptomatic carriage can result in a low-grade hemolysis.^25^ In areas where asymptomatic carriage is highly prevalent, malaria-attributable anemia can be challenging. Helminth infections are common and can exacerbate anemia while simultaneously providing a degree of protection from symptomatic malaria.^26^ However, in our study, hypereosinophilia, which is a marker of helminth infection, was not associated with a lower rate of *Plasmodium* spp. carriage. Thrombocytopenia in asymptomatic carriage was previously described in Africa.^27^ The mechanism behind thrombocytopenia may be attributed to peripheral destruction and consumption of platelets.^28^ Thus, some authors propose replacing the term “asymptomatic malaria” with “chronic malaria infection” and suggest treating all cases of *Plasmodium* spp. carriage because of their health and social consequences.^29^

### Implications for malaria transmission.

A challenge is to identify asymptomatic individuals with a level of gametocytemia sufficient to transmit infection to mosquitoes. Asymptomatic carriers could transmit malaria even at low submicroscopic parasitemias. The limits for transmission are difficult to determine because of a combination of variables, including gametocyte maturity, *Plasmodium* species, and degree of immunity. In our study, the PCR deployed to detect parasite carriers was not an ultrasensitive method. With its detection limit around parasite/µL of blood, we could assume that patients detected positive have a high probability to transmit the disease. Moreover, *P. vivax* parasites generally generate gametocytes earlier than *P. falciparum*.

### Implications for public health.

Whatever the country, *Plasmodium* spp. carriage is challenging. Control programs based on passive case detection do not detect asymptomatic infections, and, therefore, these carriers remain untreated. Because of the absence of symptoms, most infected people do not go to health facilities for testing or are under the detection limit of RDTs. Therefore, one option is to identify and treat asymptomatic patients. In our study, two remote neighborhoods represented 66.0% of the asymptomatic cases of this municipality. Such a high concentration of cases can positively influence efforts to implement control measures. Despite their distance from the STG village center, health authorities should consider these transmission hot spots if they expect better malaria control in this region. Remote-area residents were previously described as hard-to-reach populations often missed by control measures.^30^ Nonetheless, Trois-Palétuviers and Blondin 2 achieved the highest participation rates (96.3% and 80.0%, respectively). These rates suggest that these communities have a high level of concerns regarding this disease and expect action from health authorities. This highlights the importance of providing easier access to diagnosis and care in these areas. Improvements to consider include training community residents in malaria testing, as is already carried out in Brazil, and facilitating travel between Trois-Palétuviers and health centers. Finally, there are additional elimination strategies worth examining. For example, intermittent preventive treatment should be considered, in particular for certain high-risk parasite carriage populations such as Trois-Palétuviers and Blondin 2 inhabitants older than 14 years. Other strategies to consider could also include seasonal malaria chemoprevention and mass drug administration.^31–33^ However, these approaches require rigorous risk-benefit analysis before any implementation. In fact, the associated risks include an increased drug pressure that may impose selection in favor of resistance and eventual side effects in asymptomatic patients. Benefits could lead to a decreased morbidity for participants and to malaria elimination in the community. However, at this time, in France and Brazil, WHO recommendations are followed and diagnostic testing for malaria is a prerequisite to receiving treatment in symptomatic cases.^4^ Detection of asymptomatic cases is not part of the control programs. To be addressed, policymakers must commit to elimination. In addition to these asymptomatic carriage hot spots, small foci around households are observed in other neighborhoods. Generally, in low-endemic settings, reactive case detection is a viable option for febrile and RDT-positive malaria patients.^34^ In these neighborhoods, reactive case detection by PCR should be accompanied by cultural mediator-assisted discussions with the entire household. The objective is to explain the purpose of this screening and to facilitate adherence to treatment and health recommendations.

## CONCLUSION

This study allowed us to identify the main risk factors for *Plasmodium* spp. carriage in this area, where data were scare. These identified risk factors can help policymakers develop targeted intervention strategies along this border area in relation to the heterogeneous distribution. These findings may support a scientific-based choice for the future malaria program in France and Brazil, such as outreach initiatives in isolated neighborhoods that offer new points of community information, better access to malaria diagnosis, and treatment adapted to the level of transmission.
